# Ethical issues in stem cell research and therapy

**DOI:** 10.1186/scrt474

**Published:** 2014-07-07

**Authors:** Nancy MP King, Jacob Perrin

**Affiliations:** 1 Department of Social Sciences and Health Policy, Wake Forest School of Medicine, Medical Center Blvd, Winston-Salem, NC 27157, USA; 2 Center for Bioethics, Health, and Society, Wake Forest University, 1834 Wake Forest Rd, Winston-Salem, NC 27106, USA

## Abstract

Rapid progress in biotechnology has introduced a host of pressing ethical and policy issues pertaining to stem cell research. In this review, we provide an overview of the most significant issues with which the stem cell research community should be familiar. We draw on a sample of the bioethics and scientific literatures to address issues that are specific to stem cell research and therapy, as well as issues that are important for stem cell research and therapy but also for translational research in related fields, and issues that apply to all clinical research and therapy. Although debate about the moral status of the embryo in human embryonic stem cell research continues to have relevance, the discovery of other highly multipotent stem cell types and alternative methods of isolating and creating highly multipotent stem cells has raised new questions and concerns. Induced pluripotent stem cells hold great promise, but care is needed to ensure their safety in translational clinical trials, despite the temptation to move quickly from bench to bedside. A variety of highly multipotent stem cells - such as mesenchymal stem/stromal cells and stem cells derived from amniotic fluid, umbilical cord blood, adipose tissue, or urine - present the opportunity for widespread biobanking and increased access. With these increased opportunities, however, come pressing policy issues of consent, control, and justice. The imperatives to minimize risks of harm, obtain informed consent, reduce the likelihood of the therapeutic misconception, and facilitate sound translation from bench to bedside are not unique to stem cell research; their application to stem cell research and therapy nonetheless merits particular attention. Because stem cell research is both scientifically promising and ethically challenging, both the application of existing ethical frameworks and careful consideration of new ethical implications are necessary as this broad and diverse field moves forward.

## Introduction

As every reader of this journal knows, ‘stem cell research’ is a category of enormous breadth and complexity. Current and potential therapeutic applications for stem cells are numerous. Stem cell researchers may be engaged in many different endeavors, including but not limited to seeking new sources of highly multipotent stem cells and methods of perpetuating them; creating induced pluripotent stem cell (iPSC) lines to study genetic disorders or explore pharmacogenomics; conducting animal or early-phase human studies of experimental stem cell interventions; or working with stem cells and biomaterials to develop organoids and other products for use in regenerative medicine, to name only a few possibilities.

In this review of selected major ethical issues in stem cell research and therapy, we briefly describe and discuss the most significant ethical implications of this wide-ranging and fast-moving field. Our discussion addresses research oversight in the historical context of human embryonic stem cell (hESC) research; clinical translation and uncertainty; the profound tension between the desire for clinical progress and the need for scientific caution; and issues of consent, control, commercialization, and justice arising from stem cell banking, disease modeling, and drug discovery. We seek to make stem cell scientists more aware of the need for clarity of discussion and to improve professional and public understanding of the ethical and policy issues affecting this important but early research. A review this brief is necessarily general; our hope is that researchers can use this discussion as a starting point for more in-depth identification and analysis of issues pertinent to specific translational research projects [1-3].

## Stem cell research: oversight and clinical translation

The basic system of regulation and review of research involving humans and animals as subjects [4,5] is familiar to investigators. Recently, however, the term ‘translational’ has come to describe a line of research inquiry intended to stretch from bench to bedside and beyond. This has helped to emphasize that thinking about ethical issues should begin at the earliest stages of preclinical research. Ethics in both research and clinical settings is most effective when it is preventive.

In this respect, stem cell research is not unique; stem cell researchers should ask themselves the same questions about the trajectory of their translational research as would any other biomedical researcher [6]. Oversight of cell-based interventions does, however, include additional features that, while adding complexity to the regulatory process, also make it easier to take a long view, by requiring attention to the use of stem cells at all research stages. Increasing pressures for the rapid clinical translation and commercialization of stem cell products underscore the value of this long view [7-14].

The ethical issues that all researchers face during clinical translation begin with the need to ask a meaningful question, the answer to which has both scientific and social value and can be reached by the study as designed when properly conducted [6,15]. The risks of harm and the potential benefits to society from the development of generalizable knowledge (and, sometimes, potential direct benefit to patient-subjects) must be weighed and balanced at each stage of the research. Sound justification is necessary to support moving from the laboratory into animal studies, and from animals into human subjects, as well as through relevant phases of research with humans [15-18]. Minimizing the risks of harm, selecting and recruiting appropriate patient-subjects, facilitating informed decision making through the consent form and process, and avoiding the ‘therapeutic misconception’, whereby unduly high expectations affect all interested parties to a clinical trial, are all significant research ethics considerations, especially in first-in-human and other early-phase studies [19-26]. To many researchers, these considerations are simply requirements of sound and responsible study design, as exemplified, for example, in US Food and Drug Administration (FDA) guidance documents and investigational new drug requirements [27]. It should come as no surprise, however, that research design and research ethics are closely intertwined [1,6,15].

Stem cell research may give rise to heightened concern in several of these areas. One such concern is clarity of language. The term ‘stem cell’ by itself is broad and non-specific enough to be confusing; it can refer to hESCs, to iPSCs, to other types of multipotent and highly multipotent stem cells (including but not limited to stem cells derived from amniotic fluid, umbilical cord blood, adipose tissue, or urine), or to determined or adult stem cells like hematopoietic stem cells (HSCs), which have long been used in standard therapies. Patients, science reporters, and the public, on hearing the term ‘stem cell’, may thus find it difficult to distinguish between experimental stem cell interventions and proven stem cell therapies of long standing, such as treatments involving autologous or allogeneic HSC transplantation. The commercial availability worldwide of unproven ‘stem cell therapies’ that have not been studied in translational research adds to this confusion [12,14,24-26,28,29].

## Human embryonic stem cells and embryonic stem cell research oversight committees

Hopes that the ethical controversy surrounding hESCs would become irrelevant when new sources of highly multipotent stem cells became available have proven somewhat premature. hESCs remain scientifically promising and continue to have important uses, even as research with iPSCs and other highly multipotent stem cells gains momentum [30-32]. A brief discussion thus seems warranted.

The first hESC line was derived in 1998, ushering in one of the most public, spirited, and intractable debates in research ethics: the moral status of the embryo from which hESCs are derived. To harvest hESCs, it is first necessary to destroy the 5-day-old preimplantation embryo. Opponents of hESC research argue that because the embryo is capable of developing into a human being, it has significant moral standing; therefore, its destruction is unethical. Some proponents of hESC research deny that the embryo has any moral status; others grant it limited moral status but argue that the value of this limited status is far outweighed by the potential benefits that can result from hESC research [24,33].

The ethical implications of hESC research in the US have been reflected in federal funding policy and in research oversight. In 2003, the National Academy of Sciences (NAS) established a committee to develop guidelines for institutions and investigators conducting hESC research [9]. The NAS *Guidelines for Human Embryonic Stem Cell Research*, most recently amended in 2010 [34], comprehensively address permissible and impermissible categories of hESC research and recommend the establishment of embryonic stem cell research oversight committees (ESCROs) to assist in research review. They also incorporate National Institutes of Health guidelines promulgated after a 2009 federal funding expansion, recommend oversight of research with human pluripotent stem cells, and address questions of consent from all donors of biomaterials, creation and use of embryos for research purposes, and animal-human chimeras.

Many research institutions have created ESCROs or ‘SCROs’ to review hESC and iPSC research; others rely on their institutional review boards or their animal care and use committees or both. As stem cell research diversifies, its ethical oversight also becomes more diverse, and questions have been raised regarding the ongoing need for specialized committees like ESCROs and SCROs [9,10]. The NAS Guidelines are nonetheless likely to continue providing guidance for a variety of oversight bodies reviewing stem cell research [9,10,32].

## Induced pluripotent stem cells on the translational pathway

Controversy about the derivation and use of hESCs led investigators to seek less ethically fraught but maximally useful types of stem cells [31]. The history of iPSCs is one of seeking efficient ways to induce pluripotency that minimize the risk of teratoma development [35]. Although the rapidly developing science has reduced risks of harm and has increased the efficiency of pluripotent cell line creation to some extent, safety and efficacy concerns remain [36]. Indeed, the most recent advance in inducing pluripotency - stimulus-triggered acquisition of pluripotency, or STAP [37] - was widely heralded [38] but has since been called into question [39]. Obokata and colleagues [37] presented data suggesting that subjecting somatic cells to various stresses could quickly and safely produce iPSCs, but their results have not proven reproducible.

In research with iPSCs as well as with other types of stem cells, it is essential that preclinical studies in animal models and other media be sufficient to justify the progression to clinical trials. Toxicity and the risk of tumorigenicity must be assessed for all stem cell-based products, especially when genetically modified, in order to minimize the risks of harm as far as feasible before moving to humans [11,12,16,17,26,40].

Concern about the research use of animals - especially non-human primates - in preclinical research, including iPSC research, is growing and must be addressed; at the same time, researchers are increasingly aware that good animal models are often unavailable or inadequate to predict effects in humans. Thus, considerable uncertainty continues to surround first-in-human trials and other early-phase studies using stem cells, even as the rapid pace and apparently improving safety of iPSC creation tempt the field to move rapidly into clinical research and even therapeutic applications [5,25,41].

## Clinical trials: uncertainty and human subjects

Clinical trials of iPSCs and other highly pluripotent stem cell interventions generally enroll patients as subjects at all trial stages, as using healthy volunteers may raise safety concerns or compromise the value of the data. All clinical trials must, of course, be carefully designed, rigorously justified, and properly conducted in order to protect the rights, interests, and welfare of trial subjects and contribute to generalizable knowledge [11,12,15-17,25,26,35,40]. Stem cell researchers can and should benefit from the lessons learned by gene transfer researchers: rapid transition to clinical applications without sufficient understanding of the mechanisms of effect is both inefficient and unwise [11,12,25,42].

The Geron trial provides just one instructive example. In late January 2009, the FDA approved the first clinical trial of an hESC-based experimental intervention for spinal cord injury. The product, oligodendrocyte progenitor cells (OPCs), is thought to remyelinate spinal cord axons. The trial was to enroll a small number of patient-subjects with recent serious spinal cord lesions. It was placed on hold once by the FDA to ensure the purity and safety of the OPCs and ultimately was halted by the sponsor, Geron Corporation (Menlo Park, CA, USA), for reasons of cost, after only four patient-subjects had received the intervention.

Both the trial’s design and its ultimate discontinuation were controversial. Its design caused controversy because the subjects were enrolled very soon after a serious injury, making understanding and consent challenging in this first-in-human trial and in addition making it potentially difficult to distinguish between spontaneous recovery of function and remyelination attributable to the intervention. Patients with older lesions, though very probably in a better position to make decisions about trial participation, have scar tissue that makes remyelination unlikely or impossible. The sponsor’s premature discontinuation of the trial was problematic because data insufficiency renders worthless not only its own investment but also those made by patient-subjects and investigators. The outcome had the potential to discourage pioneering stem cell research in the future [25,43,44]. Nonetheless, identifying the optimal time for post-injury intervention, both to maximize the potential for assessing effects on remyelination and to promote an optimal decision-making process by patient-subjects, is of ongoing concern to spinal cord injury researchers studying cell-based interventions [45]. More recently, discussions of ethical and design issues in particular stem cell trials (for example, macular degeneration [2] and cardiovascular disease [3]) highlight the difficult balance between the imperatives of caution and progress for first-in-human trials in high-profile areas like stem cell intervention research.

Disclosure and discussion of uncertainty with potential subjects in stem cell trials are essential in order to reduce the incidence of therapeutic misconception, whereby research subjects and also investigators and oversight bodies view research as a treatment modality or significantly overestimate the likelihood of direct benefit or both [19-22,41]. This information transparency also helps protect the integrity of the research process and the safety of patients in the face of increasing global availability of unapproved and unproven stem cell ‘treatments’ [11,12,16,24-26].

Many types of multipotent and highly multipotent stem cells have been identified as potentially suitable for clinical applications. Some of the most significant challenges faced in clinical application include how quickly to move forward in the face of great promise, great uncertainty, and great clinical need; how to regard research with investigational interventions that are difficult to standardize and impossible to undo; and how to define and describe these uncertainties in the consent process. A growing number of prestigious academics from both science and bioethics are calling attention to these challenges [2,24,26,42].

One prominent scientist commentator compares the current state of stem cell research with the histories of HSC transplantation and gene transfer research, citing several principles: risks of harm should be commensurate with the severity of the condition under study, preclinical animal models remain critically important, and gaining insight into therapeutic mechanisms is essential to the success of a line of clinical research. He advocates ‘a conservative approach to clinical translation of stem cell therapies’ at present, not because of risks of harm, ‘but rather because our understanding of the mechanisms by which stem cells might prove useful, and in which diseases, remains primitive’ [25]. Similarly, in an international survey of stem cell scientists and scholars of ethical issues in stem cell research, a prolific bioethics research group has identified increasing concerns arising from pressures for clinical translation, commercialization, and oversight of new stem cell technologies [14].

## Highly multipotent stem cells: biobanking, disease modeling, and drug discovery

Some applications of stem cell research, such as disease modeling, drug discovery and testing, cell line banking, and commercialization of stem cell therapies, also give rise to ethical considerations specific to the field [11,12,14,16,24-26,28,29]. iPSCs and other highly multipotent stem cells have many additional important uses outside the typical clinical research trajectory. The creation and use of disease-specific iPSC lines, both alone and in combination with regenerative medicine products (for example, to produce *ex vivo* organoids), are essential components of disease modeling and drug discovery. ‘Body-on-a-chip’ types of three-dimensional organoid arrays hold great promise for improving drug development, disease modeling, and pharmacogenomic research, by lowering costs, speeding results, and increasing the safety and potential efficacy signaling of first-in-human trials, and considerable research is under way [46]. That promise is as yet unrealized, but questions of consent and control arise even at the bench. Because iPSC lines are derived from the somatic cells of identifiable individuals, disclosing to those individuals the planned and envisioned uses of iPSCs derived from the cells they have donated and obtaining consent from them are critical for the creation and sharing of cell line research libraries and the future uses of biomaterials derived from previously donated biospecimens [24,26,47-50].

As potentially therapeutic applications proliferate for different highly multipotent stem cell types and as technical barriers to the collection and perpetuation of cell lines continue to fall, proposed research and treatment uses abound for both autologous and allogeneic stem cells. In particular, the development of public and other broadly accessible biobanking models for stem cells derived from umbilical cord blood, amniotic fluid and placental tissue, urine, and adipose tissue holds promise for easy collection of good allograft matches for a large percentage of the population but also requires attention to ethical and policy issues [26,51].

## Justice in stem cell research and treatment

Justice is a necessary but neglected consideration in all scientific research. Like many novel biotechnologies, gene- and cell-based and regenerative medicine interventions and products can be extraordinarily costly and time- and labor-intensive to develop and use. Justice thus requires attention to the costs of developing stem cell therapies and making them available, with the goal of reducing unfair disparities in access. Cost is a standard distributive justice concern. Less commonly discussed is the effect of research funding decisions on health disparities - both priority-setting within research and priority between research funding and funding for medical care, public health, and other public goods [19-21].

Justice considerations are addressed in stem cell research and therapy in several ways. The first is biobanking policy and practice. The rationale for public stem cell banking is to provide a resource for transplantation of blood-forming HSCs to virtually anyone. Ideally, large-scale banking efforts could store enough different lines of broadly multipotent and pluripotent stem cells, suitable for use in regenerative medicine applications, to provide good matches for nearly the entire population of the US. Comprehensive systems for the collection, storage, and use of stem cells of different types are, however, still in the early stages of technological and policy development. Scientific, practical, and ethical challenges include ensuring broad availability of matches for those in need, determining access for both research and therapy, refining consent forms and processes, and protecting confidentiality in labeling and information linkage [11,12,16,26,51,52]. Thus, large-scale biobanking of stem cell lines holds the potential to greatly increase access to stem cell therapies and reduce costs, but because available allogeneic matches may not be perfect, balancing the harms and benefits of biobanking remains critical.

The second justice-promoting feature of stem cell research and therapy has some similarities. Attempts to standardize and streamline production are more prevalent in stem cell-related research than elsewhere - especially in development of cell-based products and in regenerative medicine. In other new technologies like gene transfer, standardization, the development of platform technologies, and attempts at large-scale, cost-reducing production are in their infancy. This production perspective is an important step in reducing time, labor, and costs and thus increasing access, but it could also have interesting ethical implications. Autologous or individually ‘compounded’ cell-based interventions will certainly be more costly and less readily available - and will take more time to produce - than allogeneic and other ‘mass-produced’ cell-based interventions, which may provide a less-than-perfect match or fit for a given patient. Such differences could have efficacy implications that must be monitored and balanced against cost savings and access gains [51].

A final justice consideration that is heightened in the stem cell context is the simple reality that important work dedicated to improving the health of the public takes place in a market system with its attendant pressures of competition and commercialization. The attempt to ensure that hope does not become hype and that hype does not become fraud is a matter of justice. Thus, sound practice in clinical translation, careful discussion in the media, and even seeking balance between scientific transparency and data-sharing and the intellectual property interests of industry all have important justice implications [13,16,24-29,42,53]. As research funding shrinks and competitive pressures grow, it may become increasingly difficult to move deliberately toward clinical translation and to allocate research resources wisely. This is especially likely as more is learned about how to reduce the risks of harm from the creation and use of iPSCs and as the costs of careful progress continue to increase. The fewer resources we have, the more important it is to allocate funds to maximize the likelihood of knowledge development in areas of greatest promise and clinical need [14,21].

Individual researchers may at first regard justice considerations as somewhat removed from their daily work at bench or bedside. The goals of advancing knowledge and, ultimately, improving human health are nonetheless social goals, not merely scientific goals. Researchers make vital contributions to societal views about the value of - and best directions for - scientific progress. For this reason alone, it is worthwhile for researchers to keep in mind the population-level applications of stem cell research as well as the effects of stem cell therapy on individual health.

## Summary and conclusions

As our discussion has shown, many of the ethical and policy issues that are most significant for stem cell research and therapy are similar to those arising in other novel biotechnologies. Consideration of these issues in both scientific and bioethics literatures addresses many common themes: the minimization of risks of harm; the importance of information disclosure and informed consent; the potential for overpromising, overexpectations, and the therapeutic misconception; and the pressure from disease constituencies and commercial entities to move quickly into the clinic, too often at the expense of understanding basic mechanisms. In the realm of clinical translation, trial-specific examinations of ethical issues continue to provide important guidance, not only with regard to the trials specifically considered but also as models for investigators starting down new translational pathways.

Although the creation and use of hESCs have long been the unique focus of stem cell ethics, more current controversies include the creation, for research use, of human embryos, human-animal chimeras, and gametes. Yet these marquee controversies are, in the long run, less important for the field as a whole than are more mundane, justice-oriented concerns like the creation and use of stem cell banks for research and therapy, facilitation of ‘off-the-shelf’ stem cell applications that could be less costly though perhaps less than perfect, and questions of consent, provenance, and policy. Finally, moving forward with the right blend of creativity and caution is essential, in the interest of both science and patients. In all areas of stem cell research and therapy, nuanced consideration and discussion of the best translational pathways, as viewed by ethics as well as science, will play a vital role in balancing hope and hype now and in the future, as the field continues its rapid progress.

## Abbreviations

ESCRO: embryonic stem cell research oversight (committee); FDA: US Food and Drug Administration; hESC: human embryonic stem cell; HSC: hematopoietic stem cell; iPSC: induced pluripotent stem cell; NAS: National Academy of Sciences; OPC: oligodendrocyte progenitor cell; SCRO: stem cell research oversight (committee).

## Competing interests

The authors declare that they have no competing interests.
